# Network power and mental health policy in post-war Liberia

**DOI:** 10.1093/heapol/czae020

**Published:** 2024-03-28

**Authors:** Amy S Patterson, Mary A Clark, Al-Varney Rogers

**Affiliations:** Carl Biehl Professor of International Affairs, Department of Politics, University of the South, Sewanee, TN 37375, United States; Department of Political Science, Tulane University, New Orleans, LA 70118, United States; Independent Scholar, Monrovia, Paynesville 101000, Liberia

**Keywords:** Liberia, mental health policy, key agent, network power, policy narrative

## Abstract

This article traces the influence of network power on mental health policy in Liberia, a low-income, post-conflict West African country. Based on key informant interviews, focus group discussions and document analysis, the work uses an inductive approach to uncover how a network of civil society groups, government officials, diasporans and international NGOs shaped the passage, implementation and revision of the country’s 2009 and 2016 mental health policies. With relations rooted in ties of information, expertise, resources, commitment and personal connections, the network coalesced around a key agent, the Carter Center, which connected members and guided initiatives. Network power was evident when these actors channelled expertise, shared narratives of post-war trauma and mental health as a human right, and financial resources to influence policy. Feedback loops appeared as policy implementation created new associations of mental health clinicians and service users, research entities and training institutes. These beneficiaries offered the network information from lived experiences, while also pressing their own interests in subsequent policy revisions. As the network expanded over time, some network members gained greater autonomy from the key agent. Network power outcomes included the creation of government mental health institutions, workforce development, increased public awareness, civil society mobilization and a line for mental health in the government budget, though concerns about network overstretch and key agent commitment emerged over time. The Liberian case illustrates how networks need not be inimical to development, and how network power may facilitate action on stigmatized, unpopular issues in contexts with low state capacity. A focus on network power in health shows how power can operate not only through discrete resources such as funding but also through the totality of assets that network linkages make possible.

Key messages

- Networks of public and private actors tied together through repeated exchange of information, expertise, financial resources and other assets need not be inimical to the development of health policies in contexts of low state capacity. Power manifested through embedded relations may enable networks to gain traction on stigmatized health conditions, because networks capitalize on the totality of assets their members bring to a shared policy interest. Further research will be needed to understand the circumstances that may foster non-rivalrous networks.
- Health networks may rely on key agents who facilitate communication and coordinate action among network actors. Although international NGOs may serve as key agents in low-income countries, the case of mental health in Liberia shows that they do not always exert direct influence on policymakers but rather, help network members direct their assets to achieve policy goals.
- Policy networks channel multiple assets to exert power in policy development and implementation, including expertise, a common narrative, personal experiences, strategically expended finances and formal and informal connections.

## Introduction

Liberia is a low-income West African country of about 5 million people that faces numerous health challenges (United Nations Development Programme (UNDP), 2022). During its brutal, 14-year civil war, between 60% and 77% of women suffered sexual violence (Ministry of Health & Social Welfare (MOHSW), 2009, p. 20), over 200 000 people were killed and over 1 million people were displaced. After the conflict ended in 2003, an estimated 40% of adults suffered a major depressive disorder and 44% experienced post-traumatic stress disorder (PTSD) (Johnson *et al*., 2008). Only 354 of the 550 pre-war health facilities functioned, and the one public psychiatric hospital, Catherine Mills, had been razed. Fewer than 2000 healthcare professionals remained, including only one psychiatrist, no psychologists and zero physician assistants, nurses or social workers qualified to work with psychiatric patients (Gwaikolo *et al*., 2017; Ministry of Health & Social Welfare (MOHSW), 2009, pp. 17–18). These health system deficits were evident in 2014, when the Ebola viral disease killed 10 600 people and escalated demand for mental health services (International Monetary Fund (IMF), 2022; World Health Organization (WHO)-Liberia, 2022). Years later, mental health trauma continues: the prevalence of domestic violence against girls and young women remains high (United Nations Population Fund (UNFPA), 2023); many ex-combatants and war victims suffer chronic (PTSD) (World Health Organization (WHO), 2017); and intergenerational trauma contributes to substance abuse, risky sexual behaviour and crime among youth (Interviews 9, 13, 18, 21, 28; Harris, 2008; Ministry of Health & Social Welfare (MOHSW), 2009, p. 29). A lack of care facilities, patient follow-up and medication access hamper treatment (World Health Organization (WHO)-Liberia, 2022). Despite this need, Liberia has made significant strides in mental health policy adoption, implementation and revision.

This article traces the influence of network power on Liberia’s mental health policy process. Policy networks consist of public and private actors (‘nodes’) linked by relations rooted in the exchange of information, expertise, funds, commitment and/or personal attachments (Kenis and Schneider, 1991; Wu and Knoke, 2012). Networks rest on ‘stable patterns of repeated interactions’ (Wu and Knoke, 2012, p. 154) that connect actors with shared deep core beliefs about a policy issue (Jenkins-Smith and Sabatier, 1994). Because all network actors seek resources that they alone do not have, each rationally interacts in a non-rivalrous way to achieve larger policy objectives (Compston, 2009, p. 7). Although some ‘key agents’ may have more funds, expertise and/or experiences that enable them to manage network interactions (Sørensen and Torfing, 2009), we conceptualize the network as an entity that uses its embedded relations to generate power in policy (Sriram *et al*., 2018). We define power as the ability to shape others’ actions, move issues onto or off the policy agenda and affect unconscious beliefs and internalized habits (Bachrach and Baratz, 1962; Barnett and Duvall, 2005; Buse and Hawkes, 2014; Lukes, 2018). Policy feedback loops may amplify network connections and power, because these processes give new and established actors a stake in policy implementation and revision and thus deepen the network (Campbell, 2003; Mettler, 2005).

The lens of network power provides a useful approach for analysing mental health policy development in low-income countries (LICs) such as Liberia. Wielding power through networks may be more likely to occur in LICs because weak formal institutions and limited economic resources restrict the state’s exercise of power through laws or budgets. In the absence of state power, network power builds on societal norms of reciprocity and interdependence that shape decision-making (Mkandawire, 2015; Marks and Stys, 2019). Network relations may be rooted in expertise, information and shared narratives, resources that can undergird power. The lens of network power helps to reveal how linkages among multiple actors may shape policy in LICs, where international NGOs (INGOs), bilateral and multilateral donors, state actors, civil society organizations, health experts and diasporans often interact on health governance (Swidler and Watkins, 2017).

This work strives to address some gaps in the literature on mental health policy in LICs. First, although several studies show how stigma, poor epidemiological data, doubts about cost-effective solutions, unclear outcome measures and limited funding for health are barriers to mental health policy development (Sareceno *et al*., 2007; Jenkins *et al*., 2011; Tomlinson and Lund, 2012; Gilbert *et al*., 2015; Iemmi, 2021b), they do not explain policy adoption in some LICs. Network power may play a crucial role, because state leaders often avoid highly stigmatized issues like mental health (Campbell, 2020). Second, some scholars assert that LICs’ dependence on multilateral and bilateral donor funding for health means those donors control priorities (Dionne, 2017),^1^ and since those donors have not prioritized mental health (Iemmi, 2021b), LICs do not address the issue. In Liberia, donors provided over 35% of health spending in 2019, compared to 13% from the government and over 45% from patients (Institute for Health Metrics and Evaluation (IHME), 2023). Despite this financial dependence, network power substituted for donors’ interest, affecting policy through expertise, use of narratives, strategic use of funding and consistent engagement through policy feedback loops. Liberians in government, academic institutions and civil society have been essential actors in these processes. By tracing the influence of network power in mental health policy development in Liberia, this article contributes to small collections of studies on policy networks outside the global North (Knoke, 2011), policy feedback loops in Africa (MacLean *et al*., 2016; Hern, 2019) and mental health policy in post-conflict settings (De Vries and Klazinga, 2006; Sabey, 2022).

## Methodology

Between August 2020 and May 2022, we conducted 54 semi-structured interviews and 2 focus group discussions with key informants. These included 21 interviews via Zoom and 1 by telephone in 2020 and 2021, 31 in-person interviews in summer 2022 in the capital Monrovia and 1 via Zoom that same year (see Table 1). Both focus groups were held in Monrovia: one with six Liberian mental health clinicians and one with seven school-based clinicians. Participants came from five counties (Bomi, Montserrado, Margibi, Nimba and Sinoe), with the selection criteria being geographic distribution and population representation as these counties are home to over one-half of Liberia’s population (Yates and Mehnpaine, 2023). Participants were divided roughly equally between men and women. We identified key informants purposefully, based on their past or current involvement in Liberia’s mental health programmes, and then incorporated snowball sampling to identify additional respondents. We conducted interviews until reaching saturation or that point when the replication of themes throughout the data was apparent (Morse, 1995, p. 148). INGO representatives are most numerous because of the Carter Center’s key agent role and because of the small number of government officials working in mental health. Interview questions were tailored to each key informant but the basic templates are provided in the Appendix. All respondents were given anonymity (the study collected no personal information) and promised confidentiality (the study would not reveal respondents’ identities, attach their names to quotations without permission or identify their organization if doing so could potentially reveal their identity). All study participants signed a written informed consent. Interviews lasted from 30 to 90 min; both focus groups lasted for 2 h. We conducted the focus groups seminar-style, asking open-ended questions and allowing all participants to respond. One author facilitated while others took observational notes and ensured that all members participated. Both interviews and focus groups were audio recorded, and we transcribed the recordings verbatim and then read transcripts for accuracy. For simplicity, all interviews and focus groups are numbered consecutively in one list, though the focus groups are delineated in the text.

**Table 1.** Interviews and focus groups

Number	Respondent by organizational type & position	Location	Year
1–4	INGO officials (Carter Center)	Monrovia	2022
5	Liberian Ministry of Health official (mental health unit)		
6	Liberian Ministry of Health official (administration)		
7	Liberian Ministry of Health official (information systems)		
8	Liberian Ministry of Health official (county health officer)		
9	Mother Patern College of Health (administration)		
10	Liberian Board for Nursing and Midwifery official		
11	Liberian Ministry of Education official (disability services specialist)		
12	Liberian Ministry of Gender, Children, and Social Protection official (administration)		
13	Liberian mental health advocate		
14	Liberian Police Training Academy official		
15, 16	Liberian NGO officials		
17	Focus group of six Liberian mental health practitioners		
18	Focus group of seven Liberian school counsellors trained in mental health		
19, 20	Liberian NGO officials		
21, 22	Liberian Ministry of Health officials (administration)		
23	Liberian Bureau of Immigration official		
24	INGO official (MSF)		
25	INGO official (PIH)		
26	Liberian mental health advocate		
27	INGO official (Last Mile Health)		
28, 29	Liberian NGO officials		
30	Liberian medical association official (College of Physicians and Surgeons)		
31	Liberian NGO official		
32	Liberian Ministry of Health official (regional referral hospital)		
33	Liberian Ministry of Health official (county health officer)		
34	WHO-Geneva official (Department of Mental Health)	On-line	2022
35–37	INGO officials (Carter Center)		2020
38	INGO official (Network for Empowerment and Progressive Initiative)		2021
39	INGO official (Last Mile Health)		2021
40	INGO official (International Rescue Committee)		2021
41, 42	INGO officials (Carter Center)		2021
43	INGO official (International Rescue Committee)		2021
44	US scholar (mental health specialist who volunteered in Liberia)		2020
45–47	INGO officials (Carter Center)		2020
48	Global mental health advocate (United for Global Mental Health)		2020
49, 50	INGO officials (Carter Center)		2020
51	US scholar (who consulted on Liberia’s mental health clinician training and credentialing system)		2022
52–56	INGO officials (Carter Center)	On-line; telephone	2020
**Subtotals by category** INGO officials Liberian government officials Liberian mental health advocates and NGO officials Liberian university officials Liberian medical association officials Scholars involved in Liberia’s mental health programme Global mental health advocates WHO official (Geneva) Focus groups with Liberian public sector mental health clinicians **Total**		**No. of interviews** 26 13 9 1 1 2 1 1 2 (13 persons) 56	

Note: Liberian NGOs represented include CFUH, Liberian Association of

Mental Health Practitioners, Mental Care Liberia, Liberian Mental Health Coalition, LAPS and LiCORMH.

To analyse the data, we first hand coded the transcripts using the same inductive approach for the interviews and focus groups. To increase reliability, two scholars read and coded the transcripts in an iterated fashion until themes emerged and were agreed upon. Table 2 provides examples of several themes with associated code words/phrases and illustrative quotations. We also examined documents from the Liberian government, multilateral institutions and INGOs, and we cite data from publicly available Liberian Ministry of Health, IMF, WHO, UNFPA and Carter Center documents. The authors read additional published reports from the World Bank and USAID as well as two internal Carter Center memos as background material. We used documents to establish the dates and content of policies and facts about the Liberian health system and economy, and to triangulate information from respondents.

**Table 2.** Inductive coding

Code words/phrases	Theme	Illustrative quotations
Partner, meeting, dialogue, together, discussion, help, collaboration, consensus, we decided, committee, coordinate, cooperate, work with	Network relations	‘The Technical Coordinating Committee includes international organizations, local organizations, and other government ministries. So it brings all of these people together to help advise the government through the Ministry of Health on mental health-related issues whether it is programming, whether it is a new project, or an intervention that an NGO wants to take on in the country’. (Interview 1) ‘We only work at the level of our implementing partners, they help to provide those needs that we put before them, to work with us on what we need to implement’. (Interview 5) ‘So in that policy, we decided that there would be levels of mental health … psychiatrists to be trained at the Dogliotti medical school, mid-level health workers and the Carter Center would focus on them … and we at Mother Patern would focus on social work’. (Interview 9)
Knowledge, studies, TCC gatekeeper, research, LiCORMH, Carter Center, Mother Patern, diaspora, lived experiences	Network expertise	‘We do this research, we interact with the government–Ministry of Health—provide them the findings’. (Interview 31) ‘The CFUH as that entity of persons with lived experience, we felt that they would be one of the best assessors to see if the care and treatment provided by facilities is good enough and, if not, what needs to be done to improve them’. (Interview 1) ‘In the policy review process … we’ve asked partners to share studies that may not be readily available so that we’re really drawing from the lived experience of the implementers here’. (Interview 25)
Trauma, intergenerational trauma, war, depression, anxiety, suffering, violence, drug use, addiction, risky sexual behaviour, youth	Network common narrative of post-war trauma	‘There were a lot of issues from the war. People never had the opportunity to talk to someone …. Everyone said, “Oh, the war is over! Move on!” and people went about hurting, treading a very thin line between sanity and insanity …’. (Interview 8) ‘They [children] weren’t parented during the war. … The mothers were struggling, they had to fend for themselves …. Today, those little 16 year olds, 17 year olds are having babies. They are the product of mothers who went through the war’. (Interview 12). ‘The nature of the conflict was very brutal and personal in as much as many of these people died at checkpoints in extremely arbitrary ways. So the level of trauma was visible in Liberia afterwards. If you visited Liberia afterwards you could see just walking and interacting with people the nature of the [emotional] challenge that the country was facing’. (Interview 50)
Human rights, advocacy, CFUH, lived experiences, disabilities	Network common narrative of human rights	‘They felt that we were not like them. But we fought that and said we are not beasts, we are human beings like yourself’. (Interview 15) ‘We are simply a pressure group … making sure that people with mental health conditions, psychosocial disabilities have their rights as enshrined in the International Convention on Persons with Disabilities. So we pressure the government to make sure they support mental health, as we know mental health has been for many years … under the rug’. (Interview 16) ‘But keeping people with mental illness home … does not mean you should go and lock them in chains! Or put them in the room and lock the door. No, they are human beings. They have feelings like us. They only have some mental problem, that’s all’. (Interview 26)
New organization, fund(ing), finance, establish, support civil society	Network strategic use of funds	‘We [Carter Center] provide support to Cultivation for Users’ Hope and certainly engage them in things like conferences, so there were members of Cultivation for Users’ Hope who attended the Time to Change conference with United for Global Mental Health last year’. (Interview 52) ‘And we [PIH] do whatever they ask us to do because we’re there to support them. We’re not out in front, so we have them chairing this, and we’re doing the backstopping wherever we can’. (Interview 25) ‘The model that we used is the Ethiopian public health training initiative which is at first you’re bringing in experts from the outside but they’re co-teaching with Liberians. And so by the fifth or sixth year, almost everything was being taught by the Liberian teachers and we had very few international experts’. (Interview 35)
First organization, long-term commitment, well-known, resources, coordinate, Carter Center, history	Network key agent	‘We have worked with them [the Carter Center] in looking at the policy, the Ministry of Health is now revising the mental health policy …. We went to the Carter Center many times for different meetings on different things, like curriculum, or material development, or strategy, or to share information’. (Interview 27) ‘The Carter Center is involved in many aspects, including politics. So it’s easy to influence the policy, to influence the ideology because you have contacts everywhere’. (Interview 24) ‘The Carter Center has played a pivotal role in terms of mental health awareness, which is critical. So when people are aware, then, of course, they can seek services. So that demand came from the role that the Carter Center has played here. … This is why we are now discussing mental issues, because they started to build the capacity of people [around mental health]’. (Interview 21)
Influence, attitudes, attention, resources, decline in stigma, policy outcomes, network growth	Network power	‘In the past year because of advocacy, we have the first year of a line for mental health in the government budget …. And this outcome was possible because of advocacy by the service users’. (Interview 56) ‘When Ebola happened everybody was losing their minds …. Now they [ministry experts] were seeing the importance of mental health. So it’s better; it’s getting more attention, more funding …. Now we have a psychosocial unit in the Ministry of Gender, Children, and Social Protection, and it wasn’t like that before. Now the Ministry of Health … understands mental health’. (Interview 12) ‘We … join together to help and push [mental health policy]. And that’s why we are coming together: we have the Technical Coordinating Committee …. So we are gearing up to go and just talk about it [to the legislature]. … For many of [those with mental illness], they are powerless. So where the power comes from, that’s where we need to go’. (Interview 28)
Government funding, political will, long-term commitment, sustainability, frustration	Limitations of network power	‘But the levels of support are not like they are for some of the infectious disease work …. It’s easy when you can fund a pill that you know is going to work on a person and make them better. Mental health doesn’t necessarily work that way. It can be more complicated. It can involve not only medication but also therapy, behavioural therapy and external factors and all kinds of things’. (Interview 37) ‘Under the current administration [George Weah] there have been more challenges, the priorities are elsewhere and not on the topic of mental health. The support in the line ministries is still there, but in a time of budgetary cuts, we can only do so much. If there is no money to pay people, then they cannot do the work [of mental health care]’. (Interview 56) ‘So, right now, the biggest problem has to do with resources and financing. The other thing has to do with supply of medication, there’s always shortages of psychotropic medication’. (Interview 29)

## Results

**Figure 1.**
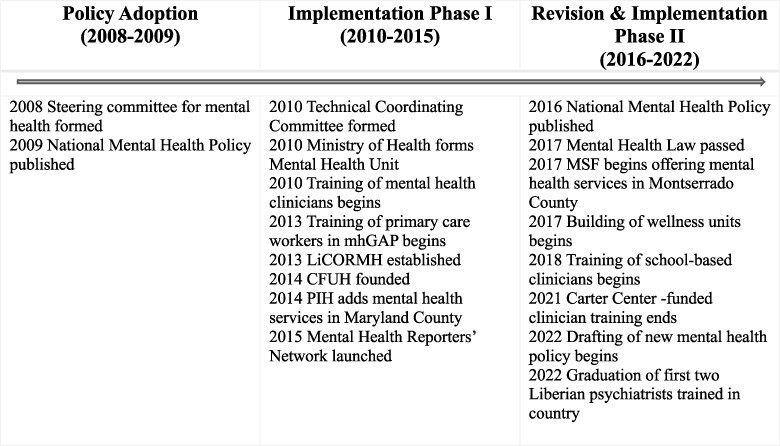
Mental health policy phases and key actions

This section first describes network nodes and their relations. The second subsection examines network power during policy adoption (2008–9) and policy implementation (2010–15), while the third subsection analyses power during policy revision and subsequent implementation (2016–22). Figure 1 provides a timeline. We do not analyse the policy revision process for an updated 2023 policy that was occurring during our fieldwork. The final subsection describes the limits of network power.

### Network nodes and relations

With diverse organizations and individuals serving as nodes, the mental health network is structured horizontally with relatively open channels of communication. Membership is broad and fluid, with Liberians in government and civil society playing crucial roles. Network boundaries are not rigid, but result from mutual recognition of the functional relevance of actors (Kenis and Schneider, 1991). Indeed, network members have portrayed some organizations as illegitimate and thus outside the network because of their work on psychosocial treatment without government approval (Interviews 9, 38, 43; Abramowitz, 2014).

The network originated with individuals who shared experiences with the war and its negative effects and who had a long-term commitment to Liberians’ well-being. Sr Barbara Brilliant, dean at the Mother Patern College of Health Sciences, a Catholic institution in Monrovia, spent most of the war in Liberia despite her expatriate status, and she worked closely with the Liberian Mr S. Benedict Dossen, head of trauma counselling at Mother Patern and since 2020, country director for the Carter Center Mental Health Program.^2^ Another Liberian, Dr Benjamin Harris served at the University of Liberia medical school until the war, which drove him to private practice. For many years he was the country’s only psychiatrist. Liberians in the diaspora who returned to rebuild their country after the war brought expertise and acted as intermediaries between local and global partners (Taslakian *et al*., 2022; Interview 41). In 2009, Dr Janice Cooper, an internationally known expert on child mental health, returned from the USA to become the first director of the Carter Center Mental Health Program. From a long-standing politically powerful Liberian family, she had intricate knowledge of Liberian politics (Cooper, 2009, p. 13). Similarly, Ms Lydia Sherman, a Liberian social worker, returned from the USA to work on mental health in the health ministry.

A significant driver for the network’s formation was the 2009 Mental Health Policy, which brought organizations into the mental health space to implement its objectives. Mandated by the policy, the Technical Coordinating Committee for Mental Health (TCC) is affiliated with the Ministry of Health and has representatives from the Mental Health Unit (see below), civil society organizations, academic and research institutions, INGOs and service user groups. An assistant minister of health and a civil society representative co-chair the TCC. The TCC reviews policy proposals, works with advocacy groups to build political support for mental health and acts as a gatekeeper to vet INGO programmes, research projects and training services to ensure fit with the mental health policy. It does not implement policies (Interviews 1, 5, 9). The TCC formally coordinates network activities, although the Carter Center has played an unofficial role as a key agent that convenes and communicates with members.

Beyond the TCC, other network nodes can be grouped by sector. In the public sector, a five-person Mental Health Unit in the Ministry of Health oversees policy implementation by public institutions and INGO partners. Illustrating network fluidity, the minister of health at times has served as a network actor who exercises authority to promote mental health, but the minister faces competing health interests and thus does not always stand with the network (Interview 12). Trained mental health clinicians, most working in public facilities, are another public node, and in 2017, many recognized their corporate professional interest and formed the Liberian Association of Mental Health Practitioners (Interview 19).

A second network grouping includes the research and academic sector. Established in 1989, the Mother Patern College of Health Sciences offers degrees in nursing, social work and biology in order to meet the country’s need for a healthcare workforce. The Liberian Center for Outcomes Research in Mental Health (LiCORMH), founded in 2013, conducts research to inform mental health practices and serves as a research repository (Interview 31). The country’s psychiatrists form another academic node, led by Harris, who in 2022 served as the president of the Liberian College of Physicians and Surgeons and the Boston University Medical Center/Boston University School of Medicine (BUMC/BUSM) Psychiatry Program.

A third grouping is Liberian civil society organizations. Registered as an NGO in 2014, Cultivation for Users’ Hope (CFUH) has over 6000 members in 6 of Liberia’s 15 counties, all of whom bring lived experiences to the policy space (Interviews 15, 16). Formed in 2008 by Liberian refugees, the Liberian Association of Psychosocial Services (LAPS) provides peer counselling and referrals primarily to survivors of war trauma and gender-based violence (Interview 29). The Mental Health Reporters’ Network of Liberia, formed in 2015, represents over 100 Liberian journalists trained to cover mental health in non-stigmatizing ways (Interview 26). From diverse local and national media outlets, they bring widely collected and distributed information to the network.

The final sectoral grouping is INGOs, who have provided technical assistance, trainings, direct funding and connections. The Carter Center Mental Health Program began in 2010, and while its staff may have had informal conversations with the government before 2010, Carter Center respondents clearly stated that they did not shape the 2009 policy and that they were invited by the Ministry of Health to help implement it (Interviews 55, 56). The INGO had worked in Liberia since 1998 on conflict resolution, democracy promotion and rule of law, enabling it to take on the role of a key agent in this nascent mental health space. It has invested in relations by sponsoring meetings and annual retreats that bring network actors together,^3^ and its 15 years of dedication to mental health impresses country partners and facilitates trust (Interviews 19, 35, 41, 42, 45, 46, 47, 50, 53, 54). Two additional INGO nodes are *Médecins Sans Frontières* (MSF), which began providing free services at five Monrovia area facilities in 2017 (Interview 24), and Partners in Health (PIH), which started providing mental health services in Maryland County in southeastern Liberia in 2014 (Interview 25).

Network relations centre around the exchange of information, expertise, shared experiences, commitment and funding, all of which undergird network power. For example, the Carter Center provides office space and internet access for LiCORMH and CFUH and training opportunities to the Reporters’ Network. In turn, those actors, as well as the Liberian Association of Mental Health Practitioners, share information about lived experiences and on-the-ground policy implementation with the Mental Health Unit and INGOs (Interviews 15, 17, 31). The health ministry uses its formal authority to initiate policy revisions and to approve INGO projects and LiCORMH research, while INGOs embed their initiatives within public facilities and LiCORMH shares its research findings with the Mental Health Unit (Interviews 10, 35). Other actors provide the ministry with needed expertise on post-war trauma (Mother Patern), workforce development (Carter Center), psychiatry (Harris and BUMC/BUSM), service delivery in LICs (PIH) and medication procurement (MSF).

### Network power in policy adoption and implementation

The initial network players (see above) gained a first victory when they successfully pushed for mental health to be included as a priority chronic disease in the 2007 National Health Plan. Inclusion ensured that mental health services would be provided free alongside other basic health services and that the government would establish a steering committee to design what became the 2009 Mental Health Policy (Lee *et al*., 2011; Interview 1). Acting before most of the aforementioned organizations emerged, these individuals faced opposition from some ‘old school’ voices in the health ministry who did not view mental health to be a priority in light of Liberia’s multiple post-war health challenges (Interview 12). The most proximate impetus for the policy occurred when Health Minister Walter Gwenigale was preparing to travel to Washington, DC, to request donor funding to rebuild the health system. Harris learned that the ministry’s proposal did not mention mental health, leading him to advocate to ministry officials for its inclusion. The donor meetings yielded significant funds to support the 2007 health plan (Hughes *et al*., 2012), and to pay consultants from the BUMC/BUSM, Massachusetts General Hospital Division of International Psychiatry, Harvard Medical School and the Harvard Program in Refugee Trauma to collect data, gather stakeholder input, research best practices and develop a situational analysis to inform the 2009 Mental Health Policy (Ministry of Health & Social Welfare (MOHSW), 2009).

The network’s power in policy adoption revolved around members’ expertise, their commitment to Liberians’ mental health and their ability to package the widespread observation about wartime trauma into a collective narrative that could catalyse action. Network actors emphasized how after the war, trauma permeated society and interpersonal distrust was high; they cited research from foreign consultants, UN agencies and domestic researchers as ‘proof’ that the population had widespread, untreated mental health problems. Research showing that people with poor mental health are less likely to support peaceful conflict resolution (Sabey, 2022) heightened concerns that Liberia could revert to war if the population could not find ‘hope for the possibility of personal and national transformation’ (Abramowitz, 2014, p. 185). This narrative resonated with both the public and policymakers, many of whom had personally experienced wartime violence and dislocation (Interview 50; Cooper, 2009). Regular communication within the network enabled its members to repeatedly convey this discourse, which prefaces the Ministry of Health’s 2009 and 2016 national mental health policies (Ministry of Health & Social Welfare (MOHSW), 2009, p. 6; Ministry of Health & Social Welfare (MOHSW), 2016, pp. 8–9).

The resulting 2009 *National Mental Health Policy* included several aspects, two of which we examine: (1) establishing the TCC, the ministry’s Mental Health Unit and LiCORMH; and (2) training primary care workers and developing a cadre of mental health clinicians (Ministry of Health & Social Welfare (MOHSW), 2009).^4^ The policy reflects conscious decisions made by Liberian stakeholders, not INGOs or bilateral or multilateral donors, such as including mental health in nationwide health services, prioritizing workforce development, deciding which workers to train and constructing institutions to support these efforts (Interview 55). Both the TCC and Mental Health Unit were established in 2010, soon after the policy’s adoption. The TCC membership included the members of the steering committee, who tapped power rooted in the experience of policy adoption to ensure broad representation on the TCC (see above) and to solidify the TCC’s role in advising the minister of health. Exercising his governmental authority, Gwenigale established the Mental Health Unit to oversee policy implementation and in so doing, became a visible champion of expanding mental health services. It was less apparent that network members from outside the ministry had a say in shaping the unit’s structure or composition, though some network INGOs funded capacity building (e.g. staff training, computers) (Interviews 5, 35).

Network power manifested through expertise and strategic use of funds to shape LiCORMH’s establishment, and LiCORMH’s subsequent research projects on the mental health aspects of Ebola, gender-based sexual violence and post-partum depression have deepened the network’s power. The Carter Center, Mother Patern, the health ministry and global North scholars generated some of LiCORMH’s first research studies. However, increased demand for psychosocial research during the Ebola outbreak transformed LiCORMH from a group dependent on these connections to an organization reliant on its own expertise and funding generated from Grand Challenges Canada and Open Society of West Africa. This transition illustrates one change in network linkages, and it highlights the autonomy and agency of a Liberian organization. LiCORMH now exercises power by sharing research findings with the Mental Health Unit to shape policy implementation (Interview 1, 31).

On the second objective, in addition to strengthening mental health knowledge among healthcare workers by training them with the WHO’s Mental Health Gap Action Programme (mhGAP) materials, the policy sought to educate nurses and physician assistants at the primary-care level to provide most mental health services (Interviews 9, 30, 41, 50, 55). Some ministry officials opposed this idea, because they thought mental healthcare providers should have years of training to diagnose illnesses and dispense medications (Interview 12). The network overcame opposition through successful policy implementation led by the Carter Center, which had expertise from its over 20 years of work on mental health and anti-stigma efforts in the USA and its 13-year health workforce development programme in Ethiopia (Carter Center, 2022; Interviews 35, 41, 47, 54). Prioritizing Liberian voices, the Carter Center worked closely with Liberian universities, the Ministry of Health, the Liberian Board for Nursing and Midwifery and the Liberian National Physician Assistants Board to design culturally appropriate curricula, to recruit healthcare workers as participants in the 6-month residential programme and to write and administer mandatory certification exams (Interviews 10, 35, 55). The Carter Center strategically used funds to support curriculum design, books, computers, transportation, consultants and faculty salaries for both foreign and Liberian instructors. Such investments deepened expertise and generated new connections for the network; these institutions also became programme beneficiaries: the Board for Nursing and Midwifery charged certification fees and the Zwedru Midwifery School was paid to host trainings (Interviews 1, 55). The policy feedback loop meant that these beneficiaries continued to push for mental health policy, and over time the network changed as these Liberian organizations became more autonomous. In 2021, Phebe Hospital and Liberian instructors, not the Carter Center, trained mental health clinicians with funding from the INGO John Snow International (Interview 2). As a result of these efforts, between 2010 and 2021, almost 500 primary-care personnel were trained with MhGAP (Interview 5; World Health Organization (WHO)-Liberia, 2016, p. 29), and 308 Liberian personnel received training and certification as mental health clinicians (168 specializing in adult mental health and 140 in child and adolescent mental health) (Carter Center, 2021). As of 2022, these clinicians served in all 15 Liberian counties, with each county having at least 18 clinicians (Interview 2). Most work in public primary-care facilities, although a few serve in dedicated mental health clinics or school-based clinics. As described below, the clinicians have a professional interest in mental health policy drawn from their experiences, and they have used their voice to affect policy revisions.

### Network power in policy revision and subsequent implementation

Policy revisions occurred in 2015, as part of a regular revision cycle in which the Ministry of Health writes an initial draft when sectoral policies expire. The TCC reviewed the ministry’s draft, a process that allowed a growing number of network actors that had directly benefited from the prior policy to influence the final outcome and agree to meet specific targets (Interview 5). Although the revised policy included several objectives (Ministry of Health & Social Welfare (MOHSW), 2016), two of the main objectives were as follows: (1) greater clinical capacity in the form of psychiatrists, wellness units and medication access; and (2) increased sensitization on mental health. These two goals reflected the network’s growing expertise in educating psychiatrists and building health capacity, its strategic use of information from beneficiaries, its transnational connections and its use of a narrative that stressed the human right to mental health care and, to a lesser extent, its messaging around intergenerational trauma (Interview 25).

By 2015, it was apparent that the country’s lack of psychiatrists was a significant problem, since psychiatrists supervise the health workers who provide most services (Ministry of Health & Social Welfare (MOHSW), 2009, p. 49) and since they diagnose and treat the most severe cases. To address this need, the network capitalized on the expertise of psychiatrists in the BUMC/BUSM group, the University of Liberia Medical School and the Liberian College of Physicians and Surgeons, as well as Harris’s years of treating Liberians. In the process, the BUMC/BUSM group became more directly involved in the network than previously when it consulted on the 2009 Mental Health Policy. Harris’s connections to other network members (e.g. Mental Health Unit) strengthened the effort to increase psychiatry education, and he served as an intermediary among BUMC/BUSM and accreditors at the University of Liberia, the Liberian College of Physicians and Surgeons and the West African College of Physicians. Committed to a philosophy of ‘bidirectional internationalism’ (Belkin and Fricchione, 2005, p. 240), the BUMC/BUSM group strategically used funds to tap a Nigerian psychiatrist to relocate to Liberia to prepare clinical facilities for a residency programme (Ghebrehiwet *et al*., 2023). PIH also strategically used funds when its programme employed a psychiatrist who could supervise medical school residents completing psychiatric rotations (Interview 24). The outcome of these efforts was that the University of Liberia Medical School began training psychiatry residents in 2019 (Interview 30). Its first psychiatrist graduated in 2022 (Global & Local Center for Mental Health Disparities (GLCMHD), 2023), adding to the two already practising in the country.

The network relied on information from the mental health clinicians and CFUH—both beneficiaries of the 2009 policy—to shape the 2016 policy objective of increased inpatient psychiatric care and provision of psychotropic medications. With inpatient care only available at the 50-bed E.S. Grant Hospital in Monrovia, the clinicians had to refer people to the capital for acute care, a great hardship for many service users. To stabilize acute cases closer to home, the policy called for establishing inpatient wellness units, at least one per county, to be run by trained clinicians at county hospitals. In addition, clinicians could not effectively treat people without medications, and in 2015, the government reported that only 7% of surveyed facilities where mental health clinicians worked had medications available (Ministry of Health & Social Welfare (MOHSW), 2016, p. 13). The network strategically used finances to promote the construction of wellness units, with five completed by 2022: two by the Carter Center, two by the Liberian government and one through a partnership between LiCORMH and the National Oil Company of Liberia (Interviews 2, 32). For medication access, MSF and PIH relied on their expertise and global reach to bargain for low-cost imported psychotropics for their facilities. MSF exercised power rooted in expertise when it organized a national study with some TCC partners to examine medication accessibility and affordability in 2022 (Interviews 24, 25).

On the second goal, Liberian network members such as the clinicians and CFUH were key in emphasizing the human rights narrative in the push for more education on mental health. These Liberian voices understood how stigma and the traditional belief that witchcraft or demonic possession caused serious mental health conditions and epileptic seizures led some affected people to suffer in silence and to avoid seeking care (Interviews 1, 3, 13, 15, 16, 17, 19, 20, 22, 33). When facilities lacked necessary medications, patients faced additional disincentives for care-seeking. As a result, demand for services was low at some facilities, making it hard for the Ministry of Health to justify finances allocated solely for mental health (Interview 22). To make the public more aware of care availability and to address misconceptions about mental illness, the clinicians educated patients’ families and community members (Interview 17 [focus group]), and CFUH conducted education and supported users through peer counselling and income-generating projects. Recognizing how culturally relevant information from lived experiences could enhance network power, the Carter Center financially supported CFUH’s establishment, but the organization quickly became autonomous. For example, it collaborated with LiCORMH and LAPS on externally funded research to examine service users’ access to legal protections (Interview 15). Additionally, the Reporters’ Network, another group that the Carter Center strategically aided with training and connections to US journalists, has published several articles to educate the public and hold the government accountable about mental health (Interviews 1, 26; see Bush Chicken, 2019). These groups capitalized on a narrative that care access and non-discrimination for the mentally ill are human rights (Interviews 15, 26). The clinicians, CFUH and the Reporters’ Network brought additional information, expertise and connections to the network, and alongside LiCORMH, illustrate changes in network relations from reliance on a few individuals and the Carter Center to increased civil society mobilization and autonomy.

Increased civil society mobilization shaped two outcomes in this policy phase. First, while we cannot quantify this perception, nearly everyone we interviewed stated that mental health issues have become more widely discussed and stigma has declined due to the work of clinicians, CFUH and the reporters (Interviews 13, 15, 16, 19). Second, in 2019, 2020 and 2021, CFUH led civil society groups to successfully advocate for a government budget line for mental health, an essential component for the long-term success of any mental health policy (Interviews 4, 9, 13, 19, 28, 46). To support this advocacy, CFUH capitalized on the network’s transnational connections to United for Global Mental Health to gain skills in political messaging and national budget analysis (Interview 48). Advocacy stressed a human rights narrative and leaned on some network members’ personal connections to policymakers in order to share testimonies during legislative hearings and private meetings with lawmakers (Interview 19). CFUH was able to exercise power because of its sizable, national membership and its legitimacy gained through lived experiences. Strategically placed and well-timed media stories from the Reporters’ Network highlighted how shortcomings in mental health services undermined patients’ fundamental human rights (Ballah, 2019; Bush Chicken, 2019). Although the outcome was only a small budgetary allocation—USD 50 000 in 2022—these efforts illustrate how civil society members have gained the strength inside and outside the network to influence policy (Interviews 5, 52).

### The limits of network power

Despite its manifestation through expertise, shared narratives, strategic financing and connections, the network’s power faced two limitations. First, it was unable to generate the level of funding needed for long-term workforce training, expanded inpatient facilities and sufficient imports of psychotropic drugs, because the government lacked the fiscal ability to allocate needed funds. In 2022, the total government budget was USD 500 million, with the Ministry of Health receiving between USD 63 million and USD 73 million. Of the ministry’s allocation, almost 70% went for personnel costs, which left only USD 20 million for all supplies, medications, facilities and infrastructure (Interview 21). In addition, major bilateral and multilateral donors had not significantly invested in mental health projects such as rebuilding the national psychiatric hospital (Interviews 19, 35, 46; see Iemmi, 2021a). Unlike some health issues such as AIDS or pandemic preparedness, mental health lacked a donor partner that could give it greater autonomy in the ministry (Interview 5). Over time, the lack of government or bilateral and multilateral donor investment affected workforce development. The mental health clinicians expressed frustration that despite their credentials, they received no pay increases. Some also felt that clinic supervisors opposed their specialization, because the supervisors did not assign them mental health cases but gave them regular nursing duties. Clinicians thought this occurred because of disregard for mental health and/or overall staff shortages [Interviews 5, 17 (focus group)]. The situation led some clinicians to establish the Liberian Association of Mental Health Practitioners in 2017 to advocate for more compensation (Interview 19), while others left public clinics to work for INGOs or USAID-funded health projects (Interview 5). Despite its expertise, shared narrative and connections, the network could do little about the clinicians’ frustrations, because the fiscal belt-tightening required for IMF loans meant the government could not increase public sector wages, regardless of network pressure to do so (Interview 8; International Monetary Fund (IMF), 2019). In addition, the network had little sway over the World Bank or USAID because those institutions’ funders and decision-makers (e.g. World Bank Executive Directors or the US Congress) were not subject to the same network pressures that Liberian actors were.

The network also had less power when some members sought to expand beyond the issues of facility-based care and community-level sensitization. In 2017, the Carter Center trained counsellors in eight secondary schools, framing the programme using the narrative of intergenerational trauma and youth mental health. The project involved collaboration with the Ministry of Education, which would support counsellors and incorporate mental health training into teacher curriculum, and the Ministry of Health, which would provide health professionals to supervise the counsellors (Interview 4). The education ministry was a partner with whom the network had limited connections and history. By 2022, 2 years after the Carter Center ended project funding, the education ministry had not designed the curriculum and the health ministry provided no supervision. Highly frustrated, school counsellors spoke of the food insecurity and domestic abuse that some students experienced, and the lack of support some counsellors felt from teachers, parents and school administrators [Interview 18 (focus group)]. The Carter Center seemed to have minimal sway over the education ministry to improve their situations (Interview 4).

## Discussion

This article has demonstrated how a horizontal, loosely organized mental health network composed of individuals, government units, civil society organizations, research bodies and INGOs was able to capitalize on members’ exchange of information, expertise and experiences, as well as shared narratives of trauma and human rights, to influence the mental health response in Liberia. The network’s power resulted in the adoption of the 2009 Mental Health Policy and its revision in 2016, actions that led to the development of a mental health workforce, increased mental health education programming and established the Mental Health Unit and LiCORMH. With an interest in these policies, civil society groups have emerged to represent service users and mental health practitioners, and these organizations have deepened network power and contributed to advocacy for mental health programmes and funding.

Mental health policy in Liberia generates some tentative lessons about network power. First, networks may play a positive role in the policy realm in LICs, where weak state capacity and opaque institutional processes may occur. Some scholars have asserted that those conditions lead networks to undermine development (van de Walle, 2001) and ‘exacerbat[e] horizontal inequality, marginalization, and corruption’ (Marks and Stys, 2019). Yet, in our specific case, network relations fostered the power needed to promote policies that provided the public good of increased mental health care and decreased stigma (Interviews 13, 15, 16, 19). Rooted in cooperation instead of rivalrous behaviour, these relations revolved around a pressing goal during the country’s post-war transition. Implementation of the 2009 policy generated positive feedback loops, as the clinicians, training institutions, Nursing Board and civil society organizations had an interest in continued policy implementation and revisions. The case also shows that as policy becomes more encompassing, the number of involved actors may grow. This increased scope may foster more legitimacy but also encourage policy drift and overreach (Ferry and Bachtler, 2013), as appeared to occur when the Carter Center embarked on the school counsellor programme.

Second, the case shows the role of a key agent in policy networks. The Carter Center provided ‘meta-governance’ in the policy process (Sørensen and Torfing, 2009), a role it could play because of its long-term commitment to Liberia, its expertise and its links to the respected Liberians in strategic positions. The network’s relatively small size enabled this key agent to centralize messaging, bring actors together and generate the reciprocities needed to work effectively (Granovetter, 1994). Yet, the role of a key agent could change with time, as was evidenced when CFUH and LiCORMH completed their own projects, received their own grants and built their own connections beyond the Carter Center. In addition, if the key agent is an INGO, it faces a project timeline and its own donor pressures, factors that could lead it to change priorities. After the Carter Center ended its school counsellor programme, some Liberian respondents worried that it would leave Liberia’s mental health space [Interview 18 (focus group)].

Third, network power may be particularly crucial for health conditions that affect marginalized populations or that have been negatively framed with moral or spiritual discourses. Governments often face little public pressure to act on those issues (Tomlinson and Lund, 2012). Network power may help actors generate and sustain interest through embedded relations that urge mutual reliance and accountability. Indeed, mobilization for access to affordable AIDS medications globally illustrates how a network of human rights groups, public health experts and people living with HIV relied on personal connections, expertise and a shared human rights narrative to challenge monopoly pricing for AIDS treatments (Kapstein and Busby, 2013).

The network power angle adds depth to the study of power in global health, because it recognizes that ties among actors matter for those actors’ ability to marshal different resources. In our analysis, power in health revolved not around access to discrete resources (i.e. expertise, funding), but rather around access to the sum of those resources that the network made possible. By illustrating ties of reliance, the network power approach provides a more accurate and nuanced portrayal of the fluid policy process. We envision that it could elucidate policy outcomes in other African countries where we have conducted research. In Ghana, for example, we could explore how power rooted in expertise has enabled a network of civil society groups, professional associations and the Mental Health Authority to push the government to pass the Mental Health Act, promote community-based mental health care and decriminalize suicide (Government of Ghana, 2012).^5^ In contrast, we could ask how limited network power in Tanzania, as evidenced by actor rivalry and poor information sharing, may explain inadequate policy implementation (Patterson, 2023).

This study has some limitations. First, because we interviewed elites in Monrovia, our findings do not reflect perspectives from throughout the country. However, because these elites decide policy, their prominence in the study is logical. In addition, recall bias could affect results, since respondents were asked to recount events of a decade (or more) ago. We triangulated data to minimize this possibility. The fact that some researchers were global North scholars may have shaped respondents’ answers, though the Liberian researcher often asked clarifying questions. Finally, our analysis does not examine long-term programme sustainability and the impact on Liberians’ mental health outcomes.

## Conclusion

This article illustrates how a network of INGOs, civil society organizations, government units, academic institutions, diasporans and service users have capitalized on their relations to exert power on mental health policy in Liberia. Network power rooted in expertise, common narratives of trauma and human rights, strategic use of funding and connections allowed actors to push for the adoption and implementation of the 2009 Mental Health Policy, an action that gave rise to additional organizations with an interest in the mental health policy’s 2016 revision and subsequent implementation. A key agent fostered network communication and shared narratives, and it used financing to support civil society and research organizations in policy implementation, groups that over time then gained autonomy. For other countries seeking to strengthen their mental health policy implementation, the Liberian case illustrates the need for an open, non-rivalrous network of actors who share expertise, narratives and commitment and whose activities a key agent with legitimacy supports and coordinates. The case also shows that host country organizations and nationals must comprise most network members, including as key agent leaders.

This article raises questions for future research on mental health policy in LICs and, more generally, network power in health. On mental health, how might the narratives that networks tap vary with context? In Liberia, the post-war trauma narrative was powerful and unquestioned, but this narrative (and its acceptance) may be highly case specific. In addition, how might the diversity of mental health conditions and the lack of well-understood aetiologies for some of them shape the network and its collaborative possibilities? If non-rivalry matters for embedded relations and reciprocities, could competition across conditions affect network power? On the broad issue of network power in health, scholars could investigate what types of key agents foster network power or the drivers of long-term network power change.

## Data Availability

Because of ethical concerns and promises of anonymity and confidentiality, the data for this project cannot be shared publicly.

## Footnotes

1. In 2019, the top multilateral and bilateral donors to Liberia were the USA, World Bank, IMF, Sweden and European Union. They provide grants directly to the government or channel money through implementing partners such as INGOs (United States Department of State and USAID, 2023); INGOs are not major donors.

2. We refer to these individuals by name because they are well-known mental health actors. This information came from secondary sources or other interviewees.

3. The authors attended one such retreat on 20 May 2022.

4. The policy also called for a mental health law, which was passed in 2017; it bars discrimination and outlines rights related to competency, guardianship, privacy and voluntary and involuntary admission and treatment (Cooper *et al*., 2021, p. 107).

5. One author conducted research with 20 mental health policy stakeholders in Ghana in 2023.

## Reflexivity statement

The authors include two women and one man. The two women are US citizens and trained political scientists: one teaches and holds an administrative position at her institution and the other is recently retired. The man is Liberian and works as a journalist and lawyer in his country. The two US scholars have engaged in research for several years on health policy and civil society mobilization, with one conducting fieldwork for extended periods in six sub-Saharan African countries and the other, in three Central American countries. Both have published reports on global mental health. The Liberian has deep knowledge of the mental health policy in his country, as well as the social and political factors shaping the policy. He also has extensive experience consulting with INGOs and donors assisting Liberia’s post-war transition.

## Appendix

### Interview templates

Questions were tailored to each key informant’s role in Liberian mental health policy. Below are the basic questions we asked all interviewees and focus group participants.

All interviews

1. How did you personally come to work in mental health?
2. How are mental disorders understood or perceived in Liberia?
3. Please describe [your institution’s] work on mental health issues in Liberia (chronology, scope).
4. Why did [your organization] become involved in [these aspects] of mental health policy?
5. (If applicable) How have you drawn on lessons or examples from other countries or entities to design your programme/activities?
6. With whom do you collaborate on these issues (individuals, organizations) and how is the work divided?
7. How do you communicate with partners and how frequently?
8. In your opinion, who are the most important actors in Liberia’s mental health field?
9. When [your organization] and its partners speak about mental health, what phrases do you use? How do you describe mental health to people?
10. To what extent have you been successful [in achieving the initiatives pursued by the organization]?
11. What have been the challenges to [these initiatives]?
12. From your perspective, how has the response to mental health disorders in Liberia changed over time?
13. Please evaluate the prospects for sustaining Liberia’s mental health programme.

Focus groups of Liberian mental health providers

1. Please describe your professional background and where you are now employed.
2. Why did you apply to be trained as a mental health clinician (clinic or school-based)?
3. Please describe the mental health conditions you see in your practice.
4. Please describe how patients, their families and the community perceive these mental health conditions.
5. Please describe the treatments you provide to patients.
6. Do you receive visits, training or other forms of supervision/continuing education from the Ministry of Health? From other sources?
7. In the course of your work, do you have contact with Cultivation for Users’ Hope or any other organization representing mental health patients or clinicians?
8. From your perspective, do the people in your catchment area have sufficient access to mental health services? Explain.
9. Is your job in mental health limited to therapeutic work or do you engage in other activities as well (e.g. community education and awareness raising)?
10. What do you regard as the successes of your work? The challenges?
11. From your perspective, how has the response to the mental health challenge in Liberia changed over time?

## References

[R1] Abramowitz SA . 2014. *Searching for Normal in the Wake of the Liberian War*. Philadelphia: University of Pennsylvania Press.

[R2] Bachrach P, Baratz M. 1962. Two faces of power. *American Political Science Review* 56: 947–52.

[R3] Ballah Z . 2019. Activists Petition Lawmakers to Prioritize Mental Health Policy in Budget. Bush Chicken. August 22. https://bushchicken.com/activists-petition-lawmakers-to-prioritize-mental-health-policy-in-budget, accessed 20 January 2024.

[R4] Barnett M, Duvall R. 2005. Power in international politics. *International Organization* 59: 39–75.

[R5] Belkin GS, Fricchione GL. 2005. Internationalism and the future of academic psychiatry. *Academic Psychiatry* 29: 240–3. 16141117 10.1176/appi.ap.29.3.240

[R6] Buse K, Hawkes S. 2014. Health post-2015: evidence and power. *The Lancet* 383: 678–9. 10.1016/S0140-6736(13)61945-5 24055453

[R7] Bush Chicken . 2019. Investigation Uncovers Patients Are Undergoing Unfair Treatment. October 31. https://www.facebook.com/watch/?v=1941460269491149, accessed 20 January 2024.

[R8] Campbell A . 2003. *How Policies Make Citizens: Senior Political Activism and the American Welfare State*. Princeton, NJ: Princeton University Press.

[R9] Campbell C . 2020. Social capital, social movements and global public health: fighting for health-enabling contexts in marginalized settings. *Social Science & Medicine* 257: 112153. 10.1016/j.socscimed.2019.02.004 30857750

[R10] Carter Center . 2021. Global Behavioral Health. https://www.cartercenter.org/health/mental_health/mh-liberia.html, accessed 1 January 2024.

[R11] Carter Center . 2022. Ethiopia Public Health Training Initiative. https://www.cartercenter.org/health/ephti/index.html, accessed 20 October 2022.

[R12] Compston H . 2009. *Policy Networks and Policy Change*. New York: Palgrave.

[R13] Cooper H . 2009. *The House at Sugar Beach: In Search of a Lost African Childhood*. New York: Simon & Schuster.

[R14] Cooper JL Gwaikolo W Thomas D . 2021. Capacity building for a mental health system of care in Liberia. In: Okpaku SO (ed). *Innovations in Global Mental Health*. Cham, Switzerland: Springer, 103–22.

[R15] De Vries AK, Klazinga NS. 2006. Mental health reform in post-conflict areas: a policy analysis based on experiences in Bosnia-Herzegovina and Kosovo. *European Journal of Public Health* 16: 246–51. 10.1093/eurpub/cki092 16524942

[R16] Dionne KJ . 2017. *Doomed Interventions: The Failure of Global Responses to AIDS in Africa*. London: Cambridge University Press.

[R17] Ferry M, Bachtler J. 2013. Reassessing the concept of policy termination: the case of regional policy in England. *Policy Studies* 34: 255–73.

[R18] Ghebrehiwet S, Ogundare T, Owusu M et al. 2023. Building a postgraduate psychiatry training program in Liberia through cross-country collaborations: initiation stages, challenges, and opportunities. *Frontiers in Public Health* 11: 1020723. 10.3389/fpubh.2023.1020723 PMC10505824 37727607

[R19] Gilbert BJ, Vikram P, Farmer PE et al. 2015. Assessing development assistance for mental health in developing countries: 2007-2013. *PLOS Medicine* 12: e1001834. 10.1371/journal.pmed.1001834 PMC4452770 26035429

[R20] Global & Local Center for Mental Health Disparities (GLCMHD) . 2023. Spotlight Interview with Dr. Moses Ziah II. https://glcmhd.org/ziah-ii-interview, accessed 19 January 2023.

[R21] Government of Ghana. 2012. Act No. 846 of 2012: Mental Health Act. https://accrapsychiatrichospital.org/new/images/downloads/Mental_Health_Act_846.pdf, accessed 14 November 2023.

[R22] Granovetter M . 1994. Business groups. In: Smelser N, Swedberg R (eds). *Handbook of Economic Sociology*. Princeton, NJ: Princeton University Press, 453–75.

[R23] Gwaikolo W, Kohrt B, Cooper J. 2017. Health system preparedness for integration of mental health services in rural Liberia. *BMC Health Services Research* 17: 508. 10.1186/s12913-017-2447-1 PMC5531097 28750617

[R24] Harris B . 2008. Substance Abuse and Sexual Behavior Assessment: High School Students in Monrovia. Monrovia, Liberia: unpublished.

[R25] Hern E . 2019. *Developing States, Shaping Citizenship: Service Delivery and Political Participation in Zambia*. Ann Arbor, MI: University of Michigan.

[R26] Hughes J, Glassman A, Gwenigale W 2012. Innovative financing in early recovery: the Liberia health sector pool fund. Working Paper 288, Washington DC: Center for Global Development.

[R27] Iemmi V . 2021a. Global collective action in mental health financing: allocation of development assistance for mental health in 142 countries, 2000–2015. *Social Science & Medicine* 287: 114354. 10.1016/j.socscimed.2021.114354 34492405

[R28] Iemmi V . 2021b. Motivation and methods of external organizations investing in mental health in low-income and middle-income countries: a qualitative study. *The Lancet Psychiatry* 8: 630–8. 33826925 10.1016/S2215-0366(20)30511-3

[R29] Institute for Health Metrics and Evaluation (IHME) . 2023. Financing Global Health: Liberia. https://vizhub.healthdata.org/fgh, accessed 20 January 2024.

[R30] International Monetary Fund (IMF) . 2019. IMF Executive Board approves US$213.6 million ECF arrangement for Liberia. Press Release, December 11. https://www.imf.org/en/News/Articles/2019/12/11/pr19451-liberia-imf-executive-board-approves-us23-4-million-ecf-arrangement#:∼:text=On%20December%2011%2C%202019%2C%20the,the%20country%20restore%20macroeconomic%20stability%2C, accessed 18 January 2024.

[R31] International Monetary Fund (IMF) . 2022. Real GDP growth- Liberia. https://www.imf.org/external/datamapper/NGDP_RPCH@WEO/LBR?zoom=LBR&highlight=LBR, accessed 5 December 2022.

[R32] Jenkins R, Baingana F, Ahmad R et al. 2011. International and national policy challenges in mental health. *Mental Health in Family Medicine* 8: 101–14. 22654973 PMC3178192

[R33] Jenkins-Smith H, Sabatier P. 1994. Evaluating the advocacy coalition framework. *Journal of Public Policy* 14: 175–203.

[R34] Johnson K, Asher J, Rosborough S et al. 2008. Association of combatant status and sexual violence with health and mental health outcomes in postconflict Liberia. *JAMA: The Journal of the American Medical Association* 300: 676–90. 18698066 10.1001/jama.300.6.676

[R35] Kapstein E, Busby J. 2013. *AIDS Drugs for All*. London: Cambridge University Press.

[R36] Kenis P Schneider V . 1991. Policy networks and policy analysis: scrutinizing a new analytical toolbox. In: Marin B, Mayntz R (eds). *Policy Networks*. Boulder, CO: Westview Press, 25–59.

[R37] Knoke D . 2011. Policy networks. In: Scott J, Carrington P (eds). *Sage Handbook of Social Network Analysis*. Thousand Oaks, CA: Sage, 210–22.

[R38] Lee PT, Kruse GR, Chan BT et al. 2011. An analysis of Liberia’s 2007 national health policy: lessons for health systems strengthening and chronic disease care in poor, post-conflict countries. *Globalization & Health* 7: 1–4. 21985150 10.1186/1744-8603-7-37 PMC3201890

[R39] Lukes S . 2018. Noumenal power: concept and explanation. *Journal of Political Power* 11: 46–55.

[R40] MacLean L, Bob-Milliar G, Baldwin E et al. 2016. The construction of citizenship and the public provision of electricity during the 2014 World Cup in Ghana. *The Journal of Modern African Studies* 54: 555–90.

[R41] Marks Z, Stys P. 2019. Research note: social network research in Africa. *African Affairs* 118: 375–91.

[R42] Mettler S . 2005. *Soldiers to Citizens: The G.I. Bill and the Making of the Greatest Generation*. London: Oxford University Press.

[R43] Ministry of Health & Social Welfare (MOHSW) . 2009. National Mental Health Policy. Monrovia, Liberia.

[R44] Ministry of Health & Social Welfare (MOHSW) . 2016. Mental Health Policy and Strategic Plan for 2016–2021. Monrovia, Liberia.

[R45] Mkandawire T . 2015. Neopatrimonialism and the political economy of economic performance in Africa: critical reflections. *World Politics* 67: 563–612.

[R46] Morse JM . 1995. The significance of saturation. *Qualitative Health Research* 5: 147–9.

[R47] Patterson A . 2023. Biomedical and spiritual approaches to mental health in Tanzania: how power and the struggle for public authority shaped care. *Studies in Comparative International Development* 58: 403–29.

[R48] Sabey C . 2022. Implementation of mental health policies and reform in post-conflict countries: the case of post-genocide Rwanda. *Health Policy & Planning* 37: 1248–56. 36062976 10.1093/heapol/czac074

[R49] Sareceno B, van Ommeren M, Batniji R et al. 2007. Barriers to improvement of mental health services in low-income and middle-income countries. *The Lancet* 370: 1164–74. 10.1016/S0140-6736(07)61263-X 17804061

[R50] Sørensen E, Torfing J. 2009. Making governance networks effective and democratic through metagovernance. *Public Administration* 87: 234–58.

[R51] Sriram V, Topp S, Schaaf M et al. 2018. 10 best resources on power in health policy and systems in low- and middle-income countries. *Health Policy & Planning* 33: 611–21. 29471544 10.1093/heapol/czy008

[R52] Swidler A, Watkins S. 2017. *A Fraught Embrace: The Romance and Reality of AIDS Altruism in Africa*. Princeton, NJ: Princeton University Press.

[R53] Taslakian E, Garber K, Shekherdimian S. 2022. Diaspora engagement: a scoping review of diaspora involvement with strengthening health systems of their origin country. *Global Health Action* 15: 2009165. 10.1080/16549716.2021.2009165 PMC8676662 34904934

[R54] Tomlinson M, Lund C. 2012. Why does mental health not get the attention it deserves? An application of the Shiffman and Smith framework. *PLoS Medicine* 9: e1001178. 10.1371/journal.pmed.1001178 PMC3289587 22389632

[R55] United Nations Development Programme (UNDP) . 2022. *Human Development Report*. https://hdr.undp.org/data-center/country-insights#/ranks, accessed 1 February 2023.

[R56] United Nations Population Fund (UNFPA) . 2023. *UNFPA Liberia Country Office Annual Report 2022*. https://liberia.unfpa.org/en/publications/unfpa-liberia-country-office-annual-report-2022, accessed 2 January 2024.

[R57] United States Department of State and USAID . 2023. Foreign Assistance by Country. https://www.foreignassistance.gov/cd/liberia/current/obligations/1, accessed 21 January 2024.

[R58] van de Walle N . 2001. *African Economies and the Politics of Permanent Crisis*. London: Cambridge University Press.

[R59] World Health Organization (WHO) . 2017. Culture and Mental Health in Liberia: A Primer. Geneva.

[R60] World Health Organization (WHO)-Liberia . 2016. Annual Report. Monrovia.

[R61] World Health Organization (WHO)-Liberia . 2022. World Mental Health Atlas 2020 Country Profile: Liberia. Geneva.

[R62] Wu CY Knoke D . 2012. Policy network models. In: Araral E et al. (ed). *Routledge Handbook of Public Policy*. London: Routledge, 153–63.

[R63] Yates DA, Mehnpaine TS. 2023. Liberia’s Population Grows by 1.7 Million People. Daily Observer (Liberia). February 23. https://www.liberianobserver.com/liberias-population-grows-17-million-people, accessed 11 January 2024.

